# Mid-infrared spectroscopy of serum, a promising non-invasive method to assess prognosis in patients with ascites and cirrhosis

**DOI:** 10.1371/journal.pone.0185997

**Published:** 2017-10-11

**Authors:** Maëna Le Corvec, Caroline Jezequel, Valérie Monbet, Nadia Fatih, Frédéric Charpentier, Hugues Tariel, Catherine Boussard-Plédel, Bruno Bureau, Olivier Loréal, Olivier Sire, Edouard Bardou-Jacquet

**Affiliations:** 1 University Bretagne Sud, IRDL, FRE CNRS 3744, Vannes, France; 2 DIAFIR, Rennes, France; 3 CHU Rennes, Liver disease unit, Rennes, France; 4 Equipe Verres et Céramiques, UMR CNRS 6226 Institut des Sciences Chimiques de Rennes, University of Rennes 1, Rennes, France; 5 IRMAR Mathematics Research Institute of Rennes, UMR-CNRS 6625, Rennes, France; 6 INRIA/ASPI, Rennes, France; 7 University of Rennes 1, Rennes, France; 8 INSERM U 1241, INRA1341, Institut NuMeCan, University of Rennes1, Rennes, France; Medizinische Fakultat der RWTH Aachen, GERMANY

## Abstract

**Background & aims:**

Prognostic tests are critical in the management of patients with cirrhosis and ascites. Biological tests or scores perform poorly in that situation. Mid-infrared fibre evanescent wave spectroscopy (MIR-FEWS) which allows for global serum metabolic profiling may provide more relevant information by measuring a wider range of metabolic parameters in serum. Here we present the accuracy of a MIR-FEWS based predictive model for the prognosis of 6 months survival in patients with ascites and cirrhosis.

**Methods:**

Patients with ascites were prospectively included and followed up for 6 months. MIR-FEWS spectra were measured in serum samples. The most informative spectral variables obtained by MIR-FEWS were selected by FADA algorithm and then used to build the MIR model. Accuracy of this model was assessed by ROC curves and 90%/10% Monte Carlo cross-validation. MIR model accuracy for 6 months survival was compared to that of the Child-Pugh and MELD scores.

**Results:**

119 patients were included. The mean age was 57.36±13.70, the MELD score was 16.32±6.26, and the Child-Pugh score was 9.5±1.83. During follow-up, 23 patients died (20%). The MIR model had an AUROC for 6 months mortality of 0.90 (CI95: 0.88–0.91), the MELD 0.77 (CI95: 0.66–0.89) and Child-Pugh 0.76 (CI95: 0.66–0.88). MELD and Child-Pugh AUROCs were significantly lower than that of the MIR model (p = 0.02 and p = 0.02 respectively). Multivariate logistic regression analysis showed that MELD (*p*<0.05, OR:0.86;CI95:0.76–0.97), Beta blockers (*p* = 0.036;OR:0.20;CI95:0.04–0.90), and the MIR model (*p*<0.001; OR:0.50; CI95:0.37–0.66), were significantly associated with 6 months mortality.

**Conclusions:**

In this pilot study MIR-FEWS more accurately assess the 6-month prognosis of patients with ascites and cirrhosis than the MELD or Child-Pugh scores. These promising results, if confirmed by a larger study, suggest that mid infrared spectroscopy could be helpful in the management of these patients.

## Introduction

Cirrhosis is associated with liver-related complications and mortality. Development of any of these complications marks the transition from a compensated to an uncompensated state which significantly decreases survival [1]. Prognosis of cirrhosis assessment is thus critical to the management of patients, especially regarding prioritization for liver transplantation. However, it remains challenging due to the wide spectrum of the consequences of liver failure and portal hypertension.

The Child-Pugh score was the first score developed to assess cirrhosis severity according to biological and clinical criteria [2,3]. Although readily available and easy to determine, it uses subjective variables that lower its reproducibility, and it does not account for renal function which has a major impact on prognosis [4]. As a result, the MELD score was developed using a robust statistical analysis [5,6]. It is based on continuous biological parameters (bilirubin, creatinine, INR), and to date, is accepted as a more reproducible scoring method, although it does not significantly outperform the accuracy of the Child-Pugh score [7]. However, unlike the Child-Pugh score, the MELD score does not account for hepatic encephalopathy or ascites, both having a critical prognostic impact [8,9].

The liver transplant waiting list recapitulates the challenges of prognosis assessment in patients with cirrhosis. The ultimate goal for each patient is to optimize the trade-off between organ availability and the risk of short-term mortality. The MELD score has been adopted to increase priority for patients with a higher risk of death [10], however MELD exception points have to be assigned to patients whose disease prognosis is not reflected by their MELD score [11]. More specifically, the onset of ascites and refractory ascites are considered as major pejorative steps not reflected by the MELD score [8]. Therefore there is still a need for a better method to assess liver disease severity, which can encompass the wide spectrum of metabolic alterations that may accompany cirrhosis, liver failure and portal hypertension.

**Fig 1:**
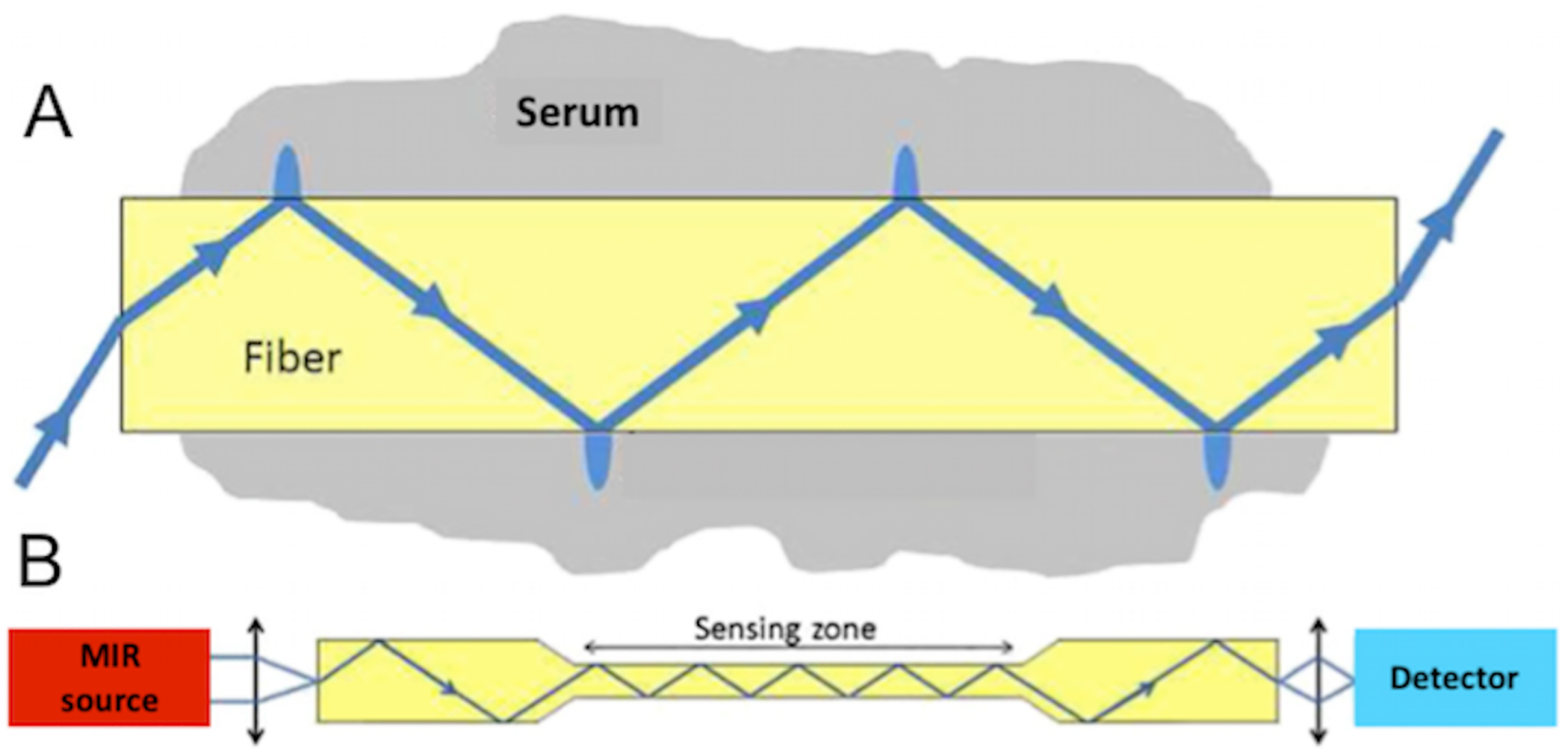
Mechanism of fibre evanescent wave spectroscopy (FEWS). A) Schematic propagation of the light wave in the fibre. B) General set up for FEWS with the light direction from Mid Infra Red (MIR) light source towards the detector. (With the agreement of Cui et al [16]).

**Fig 2:**
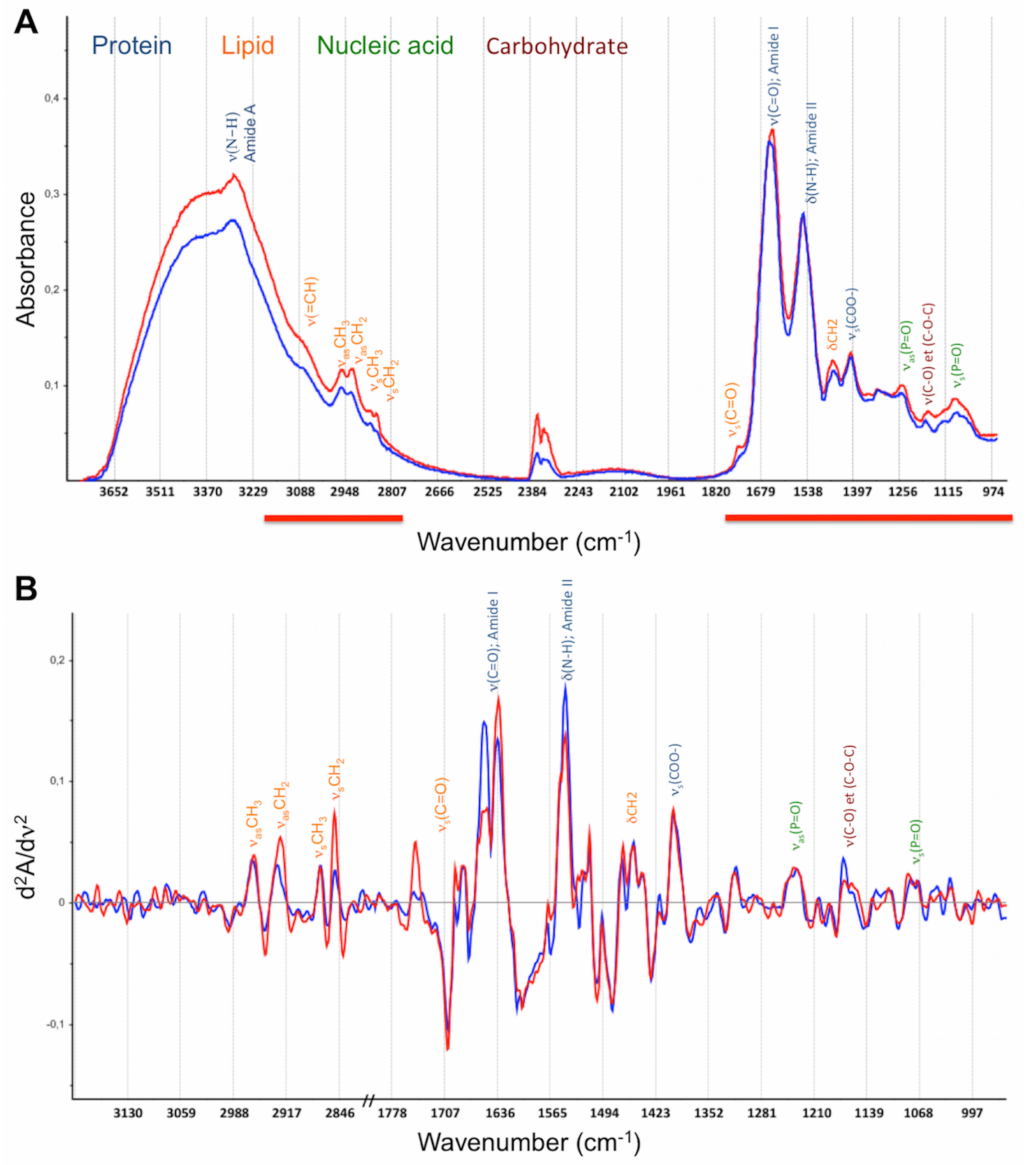
Representative spectra of a patient that was alive (blue) and a patient that was deceased (red) at 6 months. The frequency band assignments and absorption domains are displayed: blue, protein; orange, lipids; green, nucleic acids; and dark brown, carbohydrates. ν and δ symbols describe the type of vibration: ν depicts stretching and δ for bending, *as* for asymmetric and *s* for symmetric vibrational modes. A) Mid-infrared absorbance spectrum. Analysis domain (3200–2800; 1800-950cm^-1^) is displayed as red lines. B) Second derivatives spectra within the analysis domain.

Fibre Evanescent Wave Spectroscopy (FEWS) performed in the Mid Infrared (MIR) spectral domain, allows for recording of a wide metabolic profile by simply placing a drop of serum on a chalcogenide Glass fibre optic sensor and collecting an MIR absorption spectrum [12,13]. MIR spectroscopy is based on the ability of organic molecular chemical bonds to undergo vibrational transitions in the MIR region and generate absorption bands in specific and thus assignable frequency ranges [14]. Evanescent wave is the part of the electromagnetic field which propagates at the surface of a fibre when the IR beam is internally reflected at the fibre/air interface. Evanescent waves can be absorbed by the chemical groups that are in close contact with the fibre (Fig 1). Thus, putting a biological sample in contact with the fibre allows for collecting at the fibre output, the biological sample’s absorption spectrum. The spectrum reflects the whole sample’s molecular organic composition, providing a metabolic fingerprint [15] (Fig 2).

Many spectroscopy studies demonstrate the potential of MIR spectroscopy for medical diagnosis, generally from tissue [17,18] and more recently from fluids like serum [19–21], plasma [22,23], or tears [24]. In liver disease, pilot studies have unveiled the relevance of MIR spectroscopy for the diagnosis of liver or bile duct cancer [25–27]. Feasibility studies performed by our team have previously demonstrated that FEWS allows for fast and simple fluid sampling, from which relevant metabolic fingerprints can be extracted [28][29]. Despite its potential, MIR spectroscopy is not currently used in clinical practice, because most of these studies were proof of concept studies [30].

The development of medical diagnosis based on vibrational spectroscopy is a two-phase project: *i*) diagnosis or classification of biomarkers, and *ii*) identification of the collected biomarkers [31]. The metabolic fingerprint acquired from MIR spectra allows for identification of the broad biochemical alterations induced by the underlying disease. Spectral analysis and biomarker identification are required to reduce the number of spectral variables (*i*.*e*. optical densities/wavenumbers) from a few hundred to approximately a dozen, in order to minimize the influence of noisy and/or redundant variables. This can be done through projection methods (PCA or PLS), variable selection or a combination of both [32]. In biospectroscopy, most studies use projection methods, however, these methods are detrimental to the spectral assignment of discriminative variables and thus for interpretation of the model in its biochemical dimension. Furthermore, projection methods may increase both noise and over-learning risk [32], consequently,other methods were specifically designed for high-dimensional data, like the FADA algorithm [33].

The aim of this study is to evaluate the ability of MIR-FEWS to obtain a global metabolic profile useful for the assessment of short-term prognosis of patients with ascites and cirrhosis. For this purpose, the first part of this study presents the MIR spectroscopy accuracy for discriminating 6 months survival, while biochemical assignment of the selected spectral markers is proposed in the second part.

## Patients and methods

### Study population

Patients with cirrhosis and ascites, without hepatocellular carcinoma, admitted to the liver disease department between February 2012 and August 2013 for paracentesis, were consecutively included in this study. Hepatocellular carcinoma was ruled out at the first episode of ascites then screened every six months according to EASL-ECORT guidelines [34].

The study was approved by the local ethic committee (Rennes University Hospital Ethical Committee, n° 2011-A00580-41) and all patients gave their written informed consent.

History of liver disease including complications of cirrhosis, paracentesis volume and frequency, and ongoing treatment (including beta blockers, diuretics and antibiotics prophylaxis) were recorded at inclusion. A follow-up visit was performed six months after inclusion. Patients transplanted before the follow-up visit were excluded from analysis.

Clinical data, including events related to liver failure or portal hypertension were recorded at follow-up. Fasting blood samples were collected and the following biochemical assays were performed at inclusion and the follow-up visit: serum creatinine, albumin, bilirubin, prothrombin ratio, INR, sodium, AST, ALT, GGT, CRP and ALP. Child-Pugh and MELD scores were determined at inclusion and follow-up. Serum sample was frozen (-80°C) until recording for mid infrared spectra analyses.

All relevant data are available from the figshare repository at the following 10.6084/m9.figshare.4753468.

### Statistical analysis

Continuous variables were summarized as mean and standard deviation. All variables were tested for normal distribution with the Shapiro-Wilk test. Comparisons were made using the Student’s *t*-test for normally distributed data or with the Mann–Whitney U-test. Nominal data were tested using the Fisher’s exact test.

Clinically significant variables and variables associated with 6-month survival in univariate analysis with *p*<0.2, were included in logistic regression multivariate analysis with stepwise forward selection according to Wald. For multivariate analysis *p*< 0.05 was considered significant.

Diagnostic performance was determined using receiver operating characteristic (ROC) curves and comparison of ROC curves according to Delong et al [35]. Cut-offs were determined using Youden index. Data were analyzed using SPSS version 22 (SPSS Inc., Chicago, IL, USA). *p*< 0.05 was considered significant with a two-tailed test.

### Fibre evanescent wave spectroscopy

#### Spectra acquisition

The MIR spectra were recorded using a Diafir SPID^™^ FTIR portable spectrometer (Rennes, France). The FTIR spectra were acquired in absorption mode in the 4000–400 cm^-1^ frequency range. Nominal spectral resolution was set to 4 cm^-1^ and a zero filing factor of 2 was employed, yielding a discrete spectral point spacing of 2 cm^-1^. The Blackman Harris three-term apodization function was used for Fourier transform. The measurement was performed by placing a single use infrared sensor (LS-23) in the spectrometer, recording its background signal, and then depositing 7 μl of serum on the sensor. The single use design eliminates possible cross-contamination between two samples. The spectrum was acquired 5 min after serum deposition to obtain an accurate signal/water ratio and remove excess water that may conceal some infrared bands of interest. Water desorption was monitored over time by observing the 3400 cm^-1^ water νO-H absorption band decrease.

#### Pre-treatments

MIR spectra were pre-processed and analyzed in the 3800–950 cm^-1^ wavenumber domain, where most of the biomolecules absorption are located. A straight line was generated from 2800 to 1800 cm^-1^ to eliminate interference from ambient air CO_2_. Second derivatives were calculated, smoothed using a 13-point Savitzky-Golay algorithm, and normalized by vector normalization over the whole spectral range (Fig 2B). A quality test was applied and data homogeneity (outlier identification) was visually checked by principal component analysis (PCA).

#### Statistical analysis of spectra

**Fig 3:**
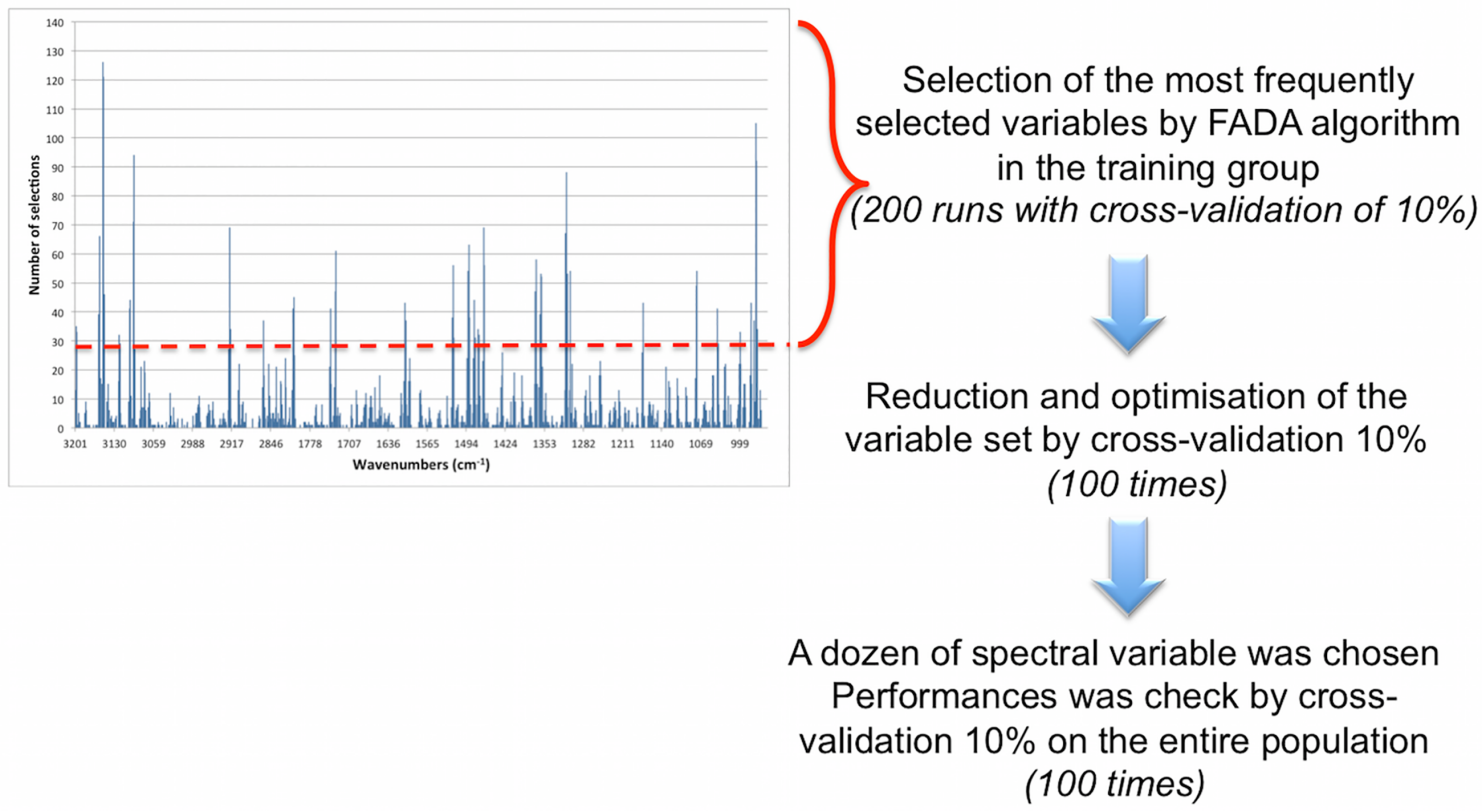
Illustrative histogram of spectral variables selection by FADA algorithm and the process of variable selection optimization for Mid-infrared predictive model construction. The red line shows the minimum for variable selection.

The statistical analyses of spectra were aimed at identifying patients at risk of death within 6 months. The wavenumber reduction process was performed only in the first 90 patients to limit the risk of overlearning. Selection of significant wavenumber within the initial set of 615 absorbance measures (3200–950 cm^-1^ with a 2800–1800 cm^-1^ gap, spectral step of 2 cm^-1^) was achieved using FADA algorithm [33]. Linear discriminant analysis (LDA) classification error rate was used as a fitness function. The variable selection process was run 200 times with a Monte Carlo cross validation of 10% [36]. The most frequently selected wavenumber (around 30) during the 200 runs were retained. Once that dataset was chosen, the LDA was run with a 90%/10% Monte Carlo cross-validation, repeated 100 times, and supervised attempts to further reduce and optimize this variable set. Once the optimized wavenumber set (a dozen) was defined, the LDA model was built using this set on the entire population and performances were checked by 90%/10% Monte Carlo cross-validation (Fig 3).

Spearman’s tests were used to study the relationship between biological parameters and spectral variables, and also between spectral variables.

All statistical analyses of spectra were performed in the R core environment [37] through packages caret [38], FADA [39] and ROCR [40].

## Results

### Patients population

One hundred and nineteen patients with cirrhosis and ascites were prospectively included. Three patients were transplanted before the follow-up visit and were excluded from analysis. Clinical and biological characteristics of patients are summarized in Table 1.

**Table 1:** Clinical and biological characteristics of patients included.

	Alive at 6 months (n = 93)	Deceased at 6 months (n = 23)	*p*
Age (years)	56.7 ± 14.4	59.9 ± 9.8	*NS*
Gender F/M	17/76	5/18	*NS*
Creatinine (mmol/l)	81.9 ± 51.2	127.2 ± 145.1	*NS*
Albumin (g/l)	30.0 ± 4.6	28.2 ± 5.1	*NS*
Bilirubin (μmol/l)	49.0 ± 54.1	144.6 ± 183.7	**
Prothrombin ratio	56.7 ± 13.6	46.6 ± 14.1	**
INR	1.58 ± 0.39	1.89 ± 0.45	**
Sodium (mmol/l)	135.8 ± 4.1	132.9 ± 3.9	**
AST (UI/l)	84.8 ±112.6	89.4 ± 52.6	*NS*
ALT (UI/l)	41.3 ± 56.5	44.3 ± 28.8	*
GGT (UI/l)	198.4 ± 184.9	255.8 ± 316.3	*NS*
CRP (mg/l)	19.7 ± 39.7	27.3 ± 24.5	**
ALP (UI/l)	151.2 ± 83.0	201.2 ± 121.1	*
MELD	14.9 ± 4.8	21.4 ± 8.2	***
Child-Pugh	9.1 ±1.7	10.9 ± 1.6	***

Values are mean ±SD.

*p<0.05.

**p<0.01.

***p<0,001 NS: non-significant.

Ninety-four (81%) patients were male with a mean age of 57.3±13.7 years. The mean MELD score was 16.32 ± 6.26 and Child-Pugh score was 9.5 ± 1.8. One hundred and nine patients had alcoholic liver disease (96%). Three patients had spontaneous bacterial peritonitis and seven presented with encephalopathy. Fifty one patients had treatment with beta blockers, 26 with furosemide, 43 with spironolactone, and 48 with spontaneous bacterial peritonitis prophylaxis.

The 6-month mortality rate was 20% (*n* = 23), and the main causes of death were liver failure (39%), sepsis (9%) and hemorrhage (13%). For 39% of patients, cause of death was either unknown or not related to liver disease. Low sodium levels and prothrombin ratio (*p*<0.01), increased INR (*p*<0.01), C-reactive protein levels (*p*<0.01), total bilirubin levels (*p*<0.01), ALT and ALP levels (*p*<0.05), and elevated MELD (*p*<0.001) and Child Pugh scores (*p*<0.001), were significantly associated with 6-month mortality (Table 1).

### Six months mortality risk assessment

All spectra passed the quality test (adjusted from [41]) and two outliers were identified by PCA analysis. These outliers were patients alive at 6 months. PCA analysis did not reveal any clear discrimination (*i*.*e*. no clustering) between deceased and not deceased patients. This prompted the use of a supervised method (LDA) with variable selection.

**Fig 4:**
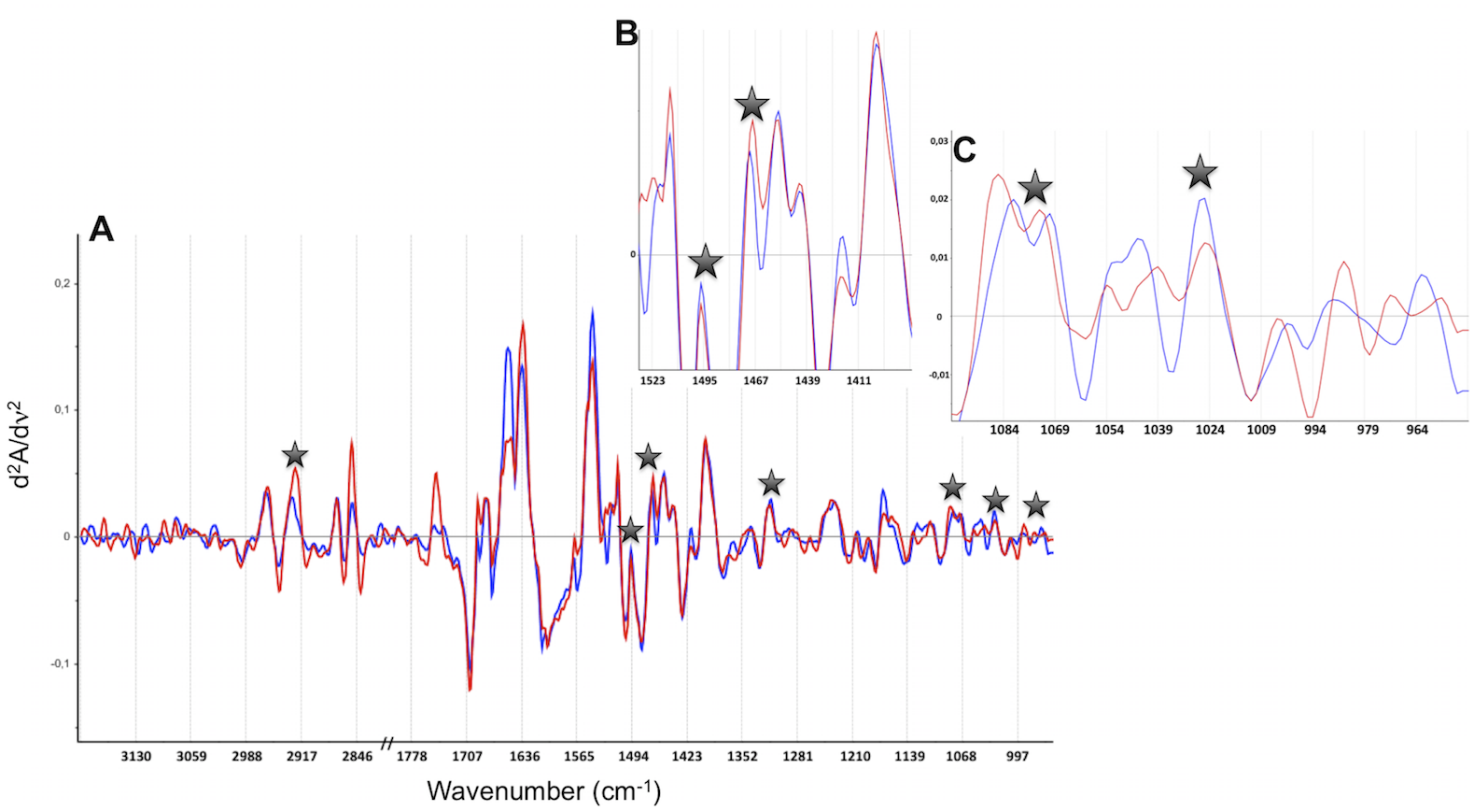
Representative spectra of one patient deceased (red) and one alive (blue) at 6 months. The seven spectral variables selected to build the predictive model are represented by stars. A) Second derivative spectra in the 3200–2800; 1800-950cm^-1^ domain. B) An expanded view in the 1530-1380cm^-1^ domain. C) An expanded view in the 1090-950cm^-1^ domain.

Representative spectra of one patient deceased and one alive at 6 months are displayed in Fig 4. Second derivative spectra showed differences between the two patients groups, but only a few of these differences were systematic. The MIR FEWS model eventually included 7 spectral variables (Table 2).

**Table 2:** Selected variable by FADA algorithm to discriminate patients alive or dead at 6 months.

Selected variables, wavenumbers (cm^-1^)	Assignments
2925	ν_as_CH_2_ acyl chains (stretching mode)
1496	Aromatic ring
1468	δCH_2_ (bending mode)
1316	Amide III, protein
1078	PO_2-_ nucleic acid / C-C and C-O endo-ring in sugars
1030	ν(C = O); RNA and exo-ring in polysaccharides
972	δ;all trans lipid

ν and δ symbols feature the type of vibration: ν for stretching and δ for bending; as for asymmetric and s for symmetric vibrational modes.

**Fig 5:**
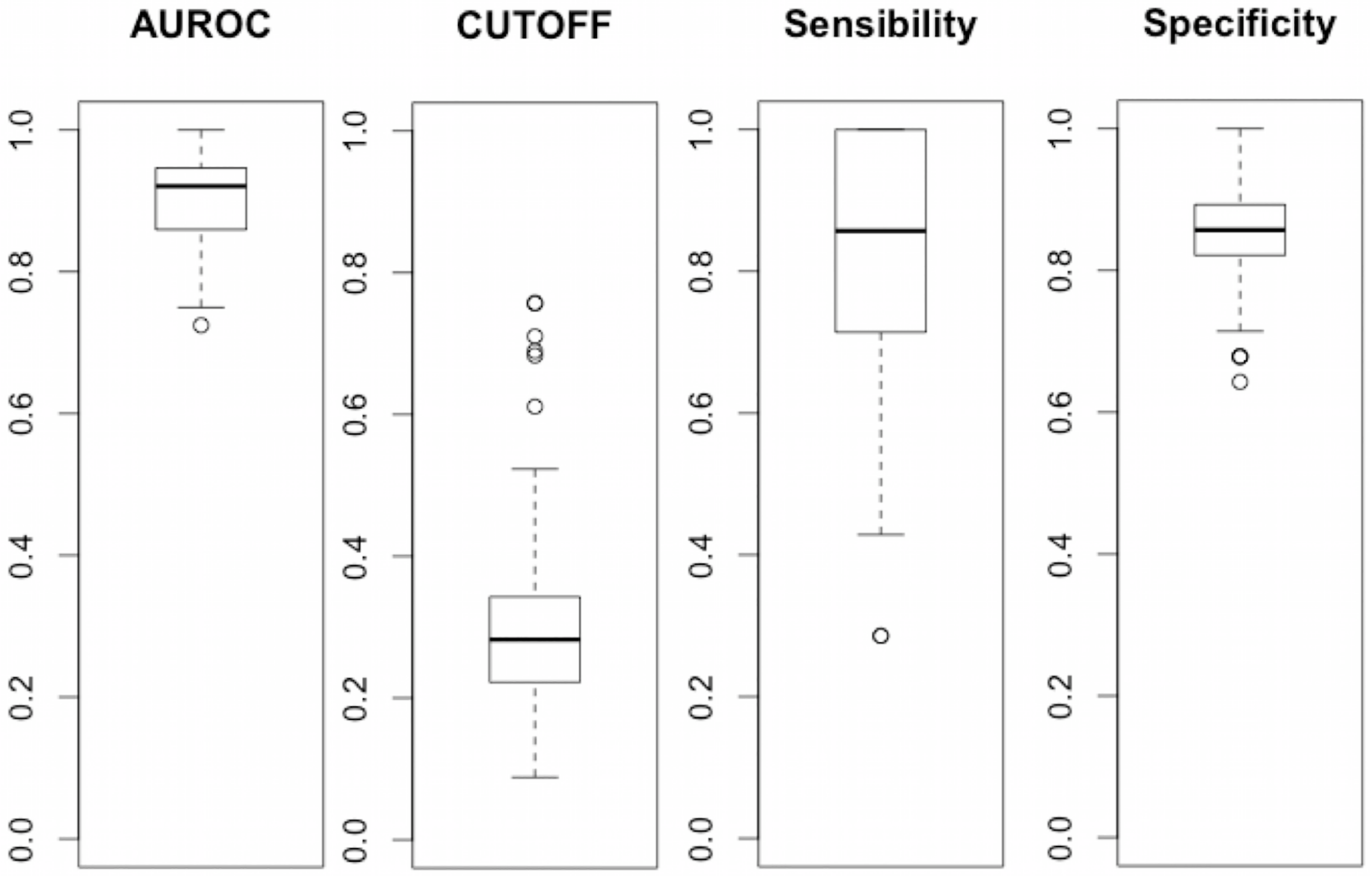
Boxplots of AUROC, cut-off, sensibility and specificity values across the 100 runs of 90%/10% Monte Carlo cross-validation for the MIR FEWS model. Boxplots of sensibility and specificity were obtained with a 0.30 cut-off value.

The mean AUROC of MIR-FEWS model over 100 cross-validation runs was 0.90 (CI95: 0.88–0.91). A mean cut-off of 0.30 was chosen to maximize sensibility and specificity. This cut-off yield a mean sensitivity of 0.79 (CI95:0.75–0.83) and specificity of 0.85 (CI95:0.84–0.87). Detailed results of the cross-validation are presented Fig 5. One deceased and six surviving patients were systematically misclassified over 100 runs of cross-validation. There was no clinical or biological evidence to explain the misclassification of these patients.

**Fig 6:**
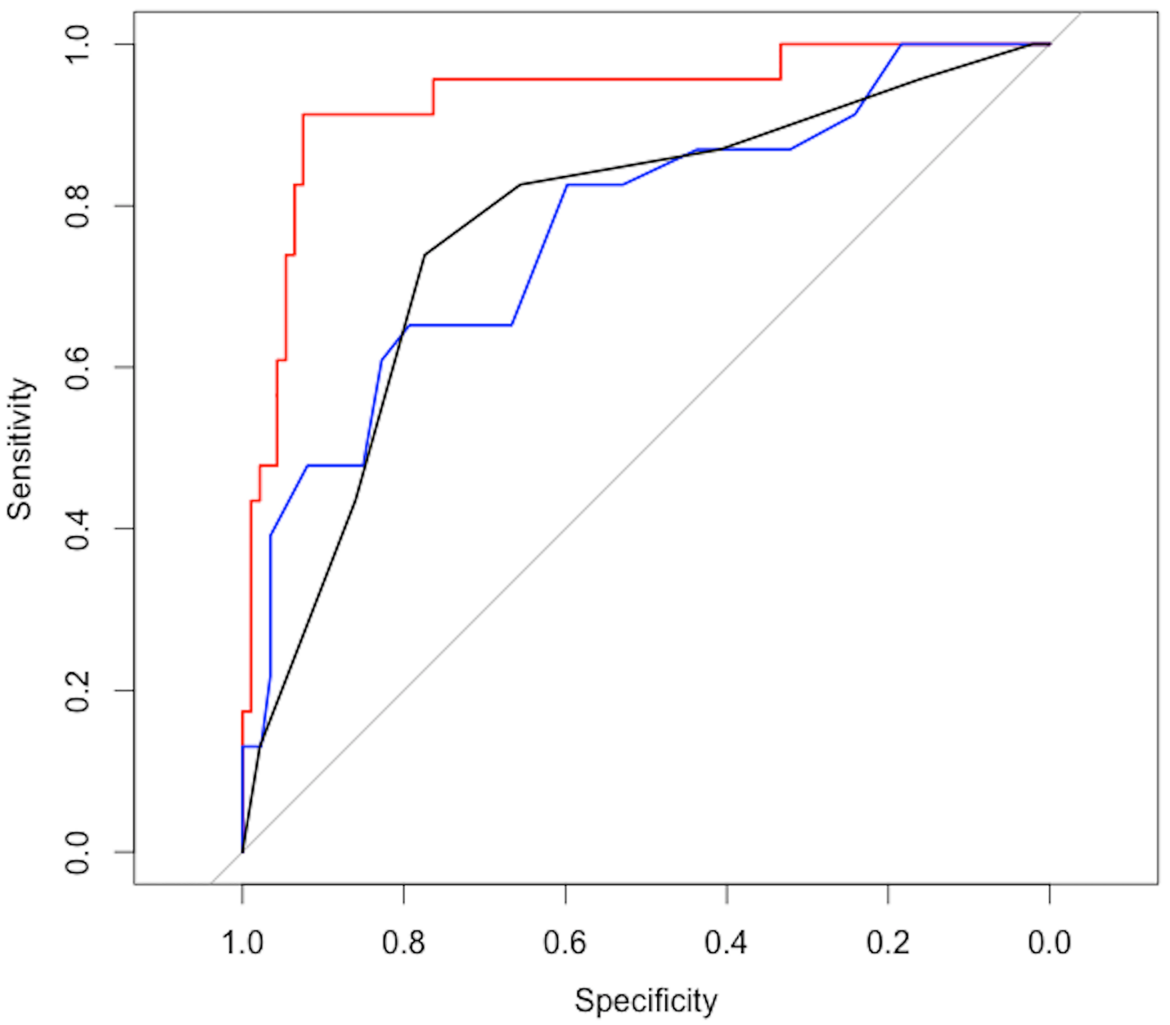
Comparison of receiver-operating characteristic (ROC) curves for the prediction of 6 months mortality. MIR model (red) (AUROC:0.94, CI95: 0.87–0.99), MELD score (blue) (AUROC:0.77, CI95: 0.66–0.89) and Child-Pugh score (AUROC: 0.76, CI95: 0.66–0.88) (black).

To compare the accuracy between the MIR model, MELD and Child-Pugh scores, the MIR model was determined for the entire population without Monte Carlo cross-validation. The AUROCs for the MIR model was 0.94 (CI95: 0.87–0.99), MELD 0.77 (CI95: 0.66–0.89) and Child-Pugh 0.76 (CI95: 0.66–0.88) (Fig 6). The AUROC of MIR-FEWS was significantly higher than that of the MELD (*p* = 0.02) and the Child-Pugh score (*p* = 0.02). There was no significant difference between the MELD and Child-Pugh AUROCs.

Logistic regression multivariate analysis identified the MELD (*p*<0.05, OR:0.86; CI95:0.76–0.97), the MIR model (*p*<0.001; OR:0.50;CI95:0.37–0.66), and beta blockers (*p* = 0.036; OR:0.20;CI95:0.04–0.90) as significant variables to explain 6-month mortality.

### Biochemical assignment of the spectral signature

Seven spectral variables (wavenumbers) were selected to build the MIR model. Chemical bonds that can be related to these wavenumbers are summarized in Table 2. These results show that 6 months mortality is associated with modifications of lipids, proteins, nucleic acids and sugar fingerprints. Concerning the lipid cluster, the 2925 cm^-1^ and 1468 cm^-1^ bands feature the CH_2_ stretching and bending modes respectively. The 972 cm^-1^ band can be assigned to the all trans lipids modes. The 1316 cm^-1^ band arises from the protein amide III vibration whereas the 1496 cm^-1^ band is associated to aromatic rings vibrations. The 1030 cm^-1^ band is specific to sugars endo-ring vibrations whereas the 1078 cm^-1^ band can arise either from PO_2_^-^ nucleic acids vibrations or from sugars exo-ring vibrations (only present in polysaccharides). Some of these spectral markers have been already identified in sera of patients exhibiting various diseases. For instance, the 1316 cm^-1^ marker was identified in sera of women exhibiting breast cancer [42], its contribution to an early diagnosis being related to significant conformation alterations in circulating proteins. The 1496 cm^-1^ band, which features aromatic rings vibrations, could be associated to nitrotyrosine–protein adducts as shown in mouse models of acetaminophen–induced hepatitis [43]. This assumption is based on the vibrational effect of the nitro group on the phenol ring vibration of Tyr amino acid residues. The 1078 cm^-1^ band is more challenging to assign. Gautam et al. [44] assigned this marker to a DNA leak in serum induced by cell necrosis.

**Fig 7:**
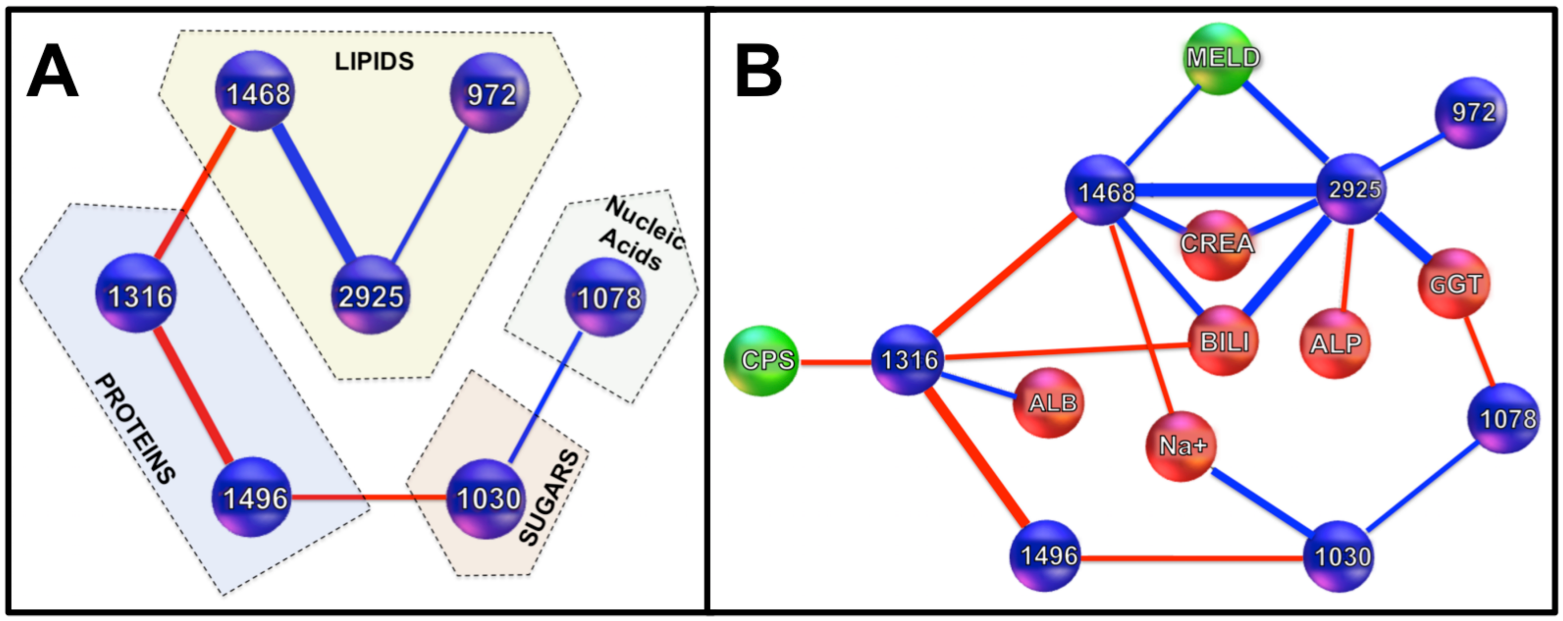
Schematic illustrations of spectral and biomolecular networks. A) Shows the network of spectral biomarkers. Rather than individual values at these 7 wavenumbers, the diagnostic prediction relies on the whole network as it is shown from the correlations between markers. Positive correlations are featured with blue edges, negative with red ones. Edge’s thickness reflects the degree of correlation (*r* value). Every correlation is significant (*p*<0.05 at least). B) Complete network of disease markers including spectral (blue), clinical and biological (red) markers and scores (green). Correlations are coded as in (A).

It is noteworthy that spectral variables are correlated one with another (Fig 7A): 2925cm^-1^ is significantly correlated with 1468cm^-1^ (*r* = 0.53, *p*<0.001) and 972cm^-1^ (*r* = 0.21, p<0.05); 1496cm^-1^ is significantly correlated with 1316cm^-1^ (*r* = -0.43, *p*<0.001) and 1030cm^-1^ (*r* = -0.22, *p*<0.05); 1468cm^-1^ is significantly correlated with 1316cm^-1^ (r = -0.26, *p*<0.01); 1078cm^-1^ is significantly correlated with 1030cm^-1^ (r = 0.19; *p*<0.05) and 972cm^-1^ (r = -0.19; *p*<0.05). It must be pointed out that these markers have been identified from second derivative spectra, therefore positive or negative correlation cannot be interpreted as similar or opposite absorbency variations. The most important point is to emphasize that the network of markers makes the recorded spectral signature and thus it is their relative variations as a whole which bear disease information.

### Linking spectral markers with clinical and biological markers

Five of the seven spectral markers were significantly correlated with biological parameters evaluated in this study (Fig 7B): 2925cm^-1^ was significantly correlated with creatinine (*r* = 0.30, *p*<0.001), bilirubin (*r* = 0.40, *p*<0.001), GGT (*r* = 0.37, *p*<0.001), ALP (*r* = 0.21, *p*<0.05), and with the MELD score (*r* = 0.31, *p*<0.01); 1468cm^-1^ with creatinine (*r* = 0.27, *p*<0.01), sodium (*r* = -0.19, *p*<0.05), bilirubin (*r* = 0.33, *p*<0.001), and with the MELD score (*r* = 0.24, *p*<0.05); 1316cm^-1^ with albumin (*r* = 0.21, *p*<0.05) bilirubin (*r* = -0.23, *p*<0.05), and with the Child-Pugh score (*r* = -0.20, *p*<0.05); 1078cm^-1^ with GGT (*r* = -0.24, *p*<0.05); and 1030cm^-1^ with sodium (*r* = -0.27, *p*<0.01).

## Discussion

Our pilot study shows that MIR-FEWS for metabolic profiling of patients with cirrhosis and ascites has a very good prognosis value. AUROC for six months mortality was 0.90(CI95: 0.88–0.91). A cut-off of 0.30 yielded a mean sensitivity of 0.79 (CI95:0.75–0.83) and specificity of 0.85 (CI95:0.84–0.87). AUROC of MELD and Child-Pugh scores were significantly lower than that of MIR model. In multivariate analyses MIR model, beta blockers treatment, and MELD scores were significantly associated with death within 6-months.

Although numerous studies have been performed to assess prognosis scores in patients with cirrhosis, few specifically assessed the prognosis of patients with ascites. In patients with cirrhosis, several studies showed similar prognosis value of the MELD and Child-Pugh scores, while others inconsistently favoured one over the other [45].

In a prospective work in patients with refractory ascites, Sersté *et al*. showed that the Child-Pugh score had a higher AUROC to predict mortality than the MELDNa score. Furthermore, severe hyponatremia was a better predictor of death than MELDNa [46]. In that study, Child-Pugh score had a higher AUROC than in our study (0.89 versus 0.76 respectively), conversely, MELD score had a lower AUROC than in our population (0.58 versus 0.77, respectively). This may be explained by differences in study populations as our population was not restricted to refractory ascites. Moreover our results were based on a 6 months restricted follow-up.

Most studies have been carried out with a 3-month follow-up and show AUROC for MELD scores and Child-Pugh of about 0.85 [47–49]. More dedicated studies performed in patients with uncompensated cirrhosis presented lower AUROC values of between 0.61 and 0.79 for the Child-Pugh and 0.76 and 0.80 for the MELD [50,51] which are consistent with our results. In order to prioritize patients for liver transplantation, MELD exception points had to be set up to offset its lack of efficiency [52]. This emphasizes the unmet need for prognosis classification in this setting.

Recent technological developments have highlighted the relevance of metabolic profiling in patients liver disease to assess their outcome [53–55]. Discrete description of metabolic alterations in serum of patients recently showed excellent performance in predicting survival of patients with uncompensated cirrhosis [56]. Although direct comparisons with available prognosis scores were not performed in this study, it supports the potential benefit of metabolic profiling. Limitations of the method used in that study include its complexity, expensive equipment and analyses required to implement these metabolomics tests. Our study uses a simple benchtop method which, although not giving a definite picture of the various metabolic alterations, provides a global fingerprint that shows satisfactory prognosis assessment. The pattern approach used in our study emphasizes the relevance of relative variations in identified spectral markers, which is facilitated by the fact that MIR spectra feature a wide range of relevant metabolites.

In conclusion, compared with the MELD and Child-Pugh scores, the MIR-FEWS method appears to be more efficient at identifying patients at risk for short-term mortality. This could be related to the ability of MIR-FEWS to obtain global information on the metabolic status of patients, in contrast to conventional scores which are based upon a limited number of parameters. The relevance of MIR-FEWS should be confirmed in an independent and large cohort of patients with cirrhosis and ascites. In addition, our study suggests the relevance of using the MIR-FEWS method in patients with cirrhosis with or without ascites.

## Abbreviations

ALP: alkaline phosphatase
ALT: alanine aminotransferase
AST: aspartate aminotransferase
AUROC: area under the receiver operating characteristic curve
CRP: C-reactive protein
FEWS: fibre evanescent wave spectroscopy
FTIR: Fourier transform infrared
GGT: γ-glutamyltransferase
INR: International Normalized Ratio
LDA: linear discriminant analysis
MELD: model for end-stage liver disease
MIR: mid-infrared
PCA: principal component analysis
PLS: partial least squares
ROC: receiver-operating characteristic
